# Biodegradable and Toughened Composite of Poly(Propylene Carbonate)/Thermoplastic Polyurethane (PPC/TPU): Effect of Hydrogen Bonding

**DOI:** 10.3390/ijms19072032

**Published:** 2018-07-13

**Authors:** Dongmei Han, Guiji Chen, Min Xiao, Shuanjin Wang, Shou Chen, Xiaohua Peng, Yuezhong Meng

**Affiliations:** 1 The Key Laboratory of Low-carbon Chemistry & Energy Conservation of Guangdong Province/State Key Laboratory of Optoelectronic Materials and Technologies, Sun Yat-Sen University, Guangzhou 510275, China; handongm@mail.sysu.edu.cn (D.H.); chenguiji@kingfa.com.cn (G.C.); stsxm@mail.sysu.edu.cn (M.X.); 2 School of Chemical Engineering and Technology, Sun Yat-Sen University, Guangzhou 510275, China; 3 Shanghai Kingfa Science and Technology Development Co., Ltd., Shanghai 201714, China; 4 Shenzhen Beauty Star Co., Ltd., Shenzhen 518112, China; chens@beautystar.cn (S.C.); alice@beautystar.cn (X.P.)

**Keywords:** Poly(propylene carbonate), thermoplastic polyurethane, compatibility, toughness

## Abstract

The blends of Poly(propylene carbonate) (PPC) and polyester-based thermoplastic polyurethane (TPU) were melt compounded in an internal mixer. The compatibility, thermal behaviors, mechanical properties and toughening mechanism of the blends were investigated using Fourier transform infrared spectra (FTIR), tensile tests, impact tests, differential scanning calorimetry (DSC), scanning electron microscopy (SEM) and dynamic mechanical analysis technologies. FTIR and SEM examination reveal strong interfacial adhesion between PPC matrix and suspended TPU particles. Dynamic mechanical analyzer (DMA) characterize the glass transition temperature, secondary motion and low temperature properties. By the incorporation of TPU, the thermal stabilities are greatly enhanced and the mechanical properties are obviously improved for the PPC/TPU blends. Moreover, PPC/TPU blends exhibit a brittle-ductile transition with the addition of 20 wt % TPU. It is considered that the enhanced toughness results in the shear yielding occurred in both PPC matrix and TPU particles of the blends.

## 1. Introduction

Poly(propylene carbonate) (PPC) is a biodegradable aliphatic polycarbonate derived from carbon dioxide and propylene oxide. It has been paid much attention because of its high value-added fixation of CO_2_, biodegradability and excellent oxygen barrier performance [1,2,3]. However, because PPC exhibits amorphous nature and possess low glass-transition temperature (Tg), it becomes brittle at low temperature and quickly loses strength at elevated temperature, which severely limits its wide application [4]. Many efforts have been devoted to overcoming these drawbacks. Chemical methods such as terpolymerization of carbon dioxide and propylene oxide with another monomer—for example, cyclohexene oxide (CHO) [5], maleic anhydride [6], or caprolactone [7]—have been employed. Simultaneously, physical blending of PPC with other polymers remains attractive because it is simple and cost-effective. Several PPC-base alloys have been prepared, starch [8,9], poly(ε-hydroxybutyrate-co-ε-hydroxyvalerate) (PHBV) [10], poly(3-hydroxybutyrate) (PHB) [11], poly(lactic acid) (PLA) [12], poly(butylene succinate) (PBS) [13], poly(ethylene-co-vinyl alcohol) (EVOH) [14] or others have been used to modify PPC. Nevertheless, most of these blends exhibited low miscibility between each component due to relatively weak inter-molecular interactions. For this reason, toughening of PPC is rarely achieved.

Thermoplastic polyurethane (TPU) elastomer, a linear segmented block copolymer consist of alternating soft segments and hard segments, are extensively used due to its superior properties, including high strength, high toughness, abrasion resistance, low temperature flexibility, biocompatibility, durability, biostability and so forth. [15,16,17]. Several works have been reported that TPU can be used as flexibilizer in many brittle polymers, such as polypropylene [18], poly(lactic acid) [19,20], polyacetal [21], poly(butylene terephthalate) [22] and so on.

**Scheme 1:**
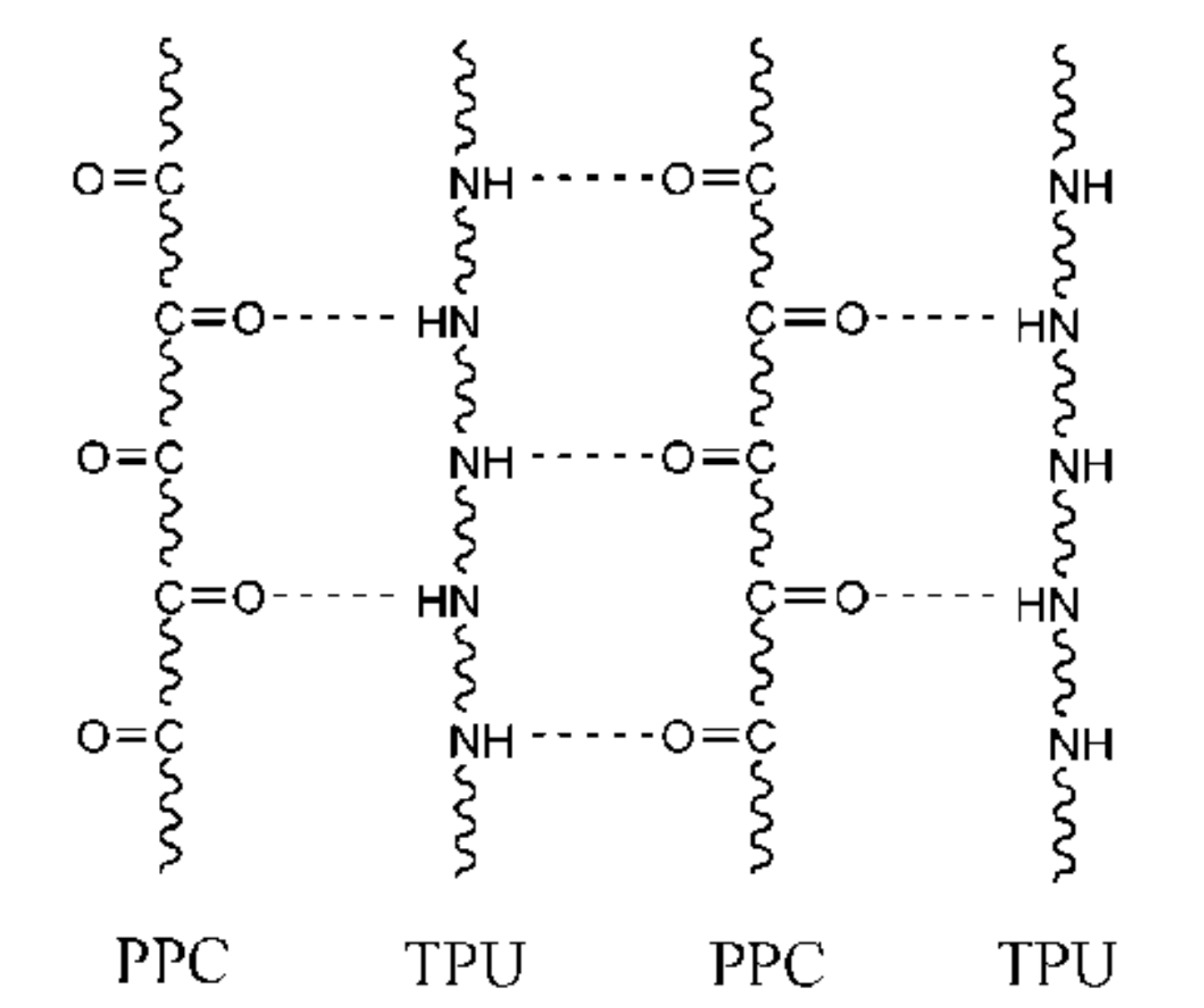
Schematic illustration of hydrogen-bonding between the molecules of PPC and TPU.

Because of the similarly chemical structure, it is believed that PPC and TPU have better miscibility. Therefore, according to the phenomena that PPC becomes brittle at low temperature and loses its strength rapidly at high temperature, thermoplastic polyurethanes (TPU) are used to toughen PPC, in this work. Furthermore, the urethane moiety in hard segment of TPU can form hydrogen bonding with the carbonyl groups in PPC (Scheme 1), which in turn increase the interaction of molecular chain between PPC and TPU. In these connections, PPC was blended with TPU is expected to improve its toughness and thermal stability. These improvements will broaden the practical application of the new biodegradable PPC.

## 2. Results and Discussion

### 2.1. FTIR Investigation

**Figure 1:**
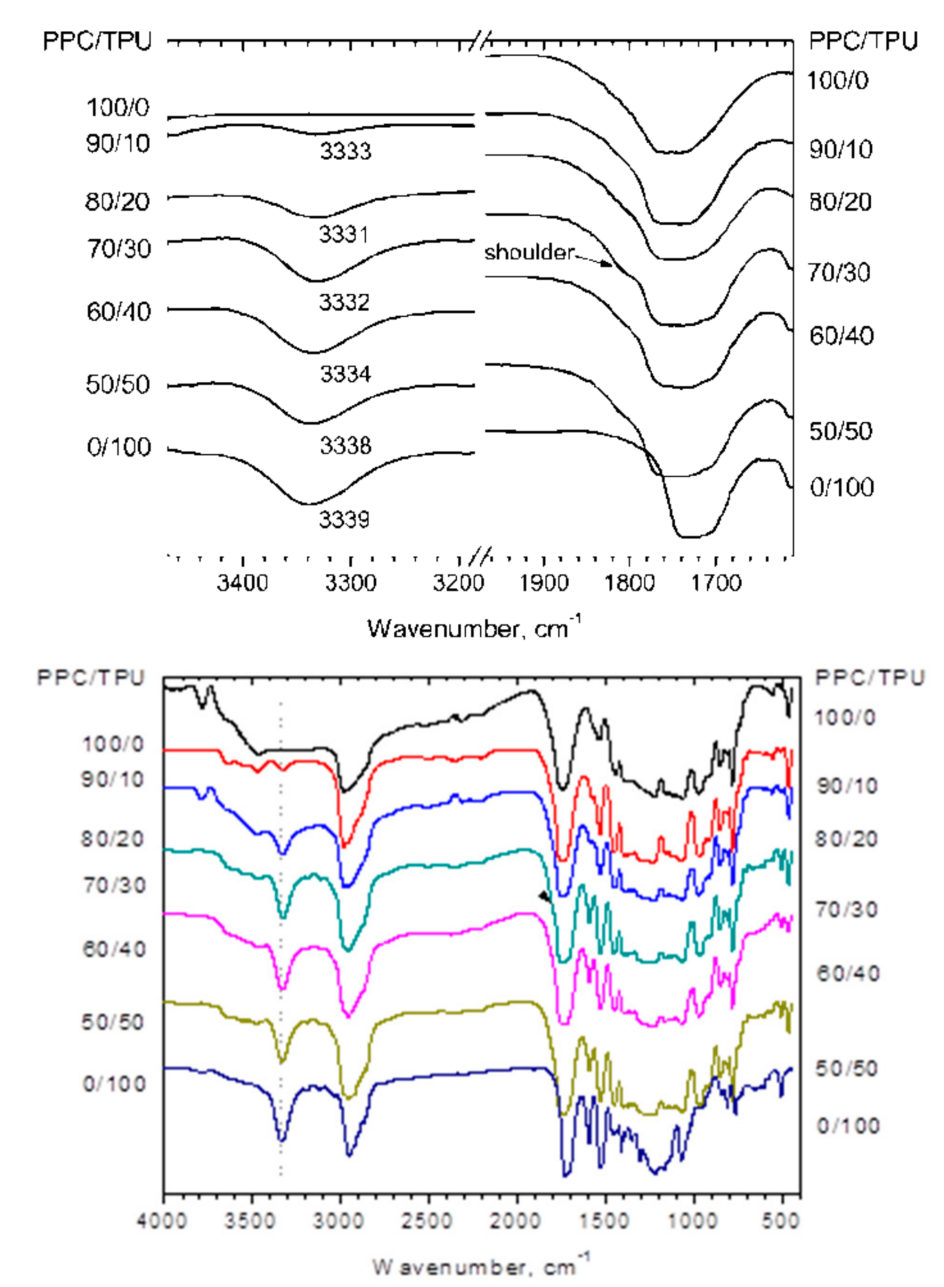
FTIR spectra of neat PPC, neat TPU and PPC/TPU blends.

It is well known that the interaction between two components of the blend can be identified with FTIR technology. If two polymers are compatible, the absorption band shifts and broadening in the FTIR spectra indicates a chemical interaction between the molecular chains of one polymer and the other [23]. It was reported that there are the hydrogen-bonding interactions in their blends between Poly(propylene carbonate) (PPC) and other polymers, such as starch [8] and poly(ethylene-co-vinyl alcohol) (EVOH) [14]. Similarly, FTIR technique was used to confirm the existence of molecular chain interaction between the PPC and TPU in the blends. FTIR spectra of neat PPC, neat TPU and PPC/TPU blends with different TPU content are shown in Figure 1. The C=O absorption peak at 1749 cm^−1^ and N–H absorption peak at 3339 cm^−1^ were observed in the neat PPC and TPU respectively. In the blends, with the increase of PPC content, the N–H peak shifted towards lower wavenumber in the presence of PPC. It indicates a certain intermolecular hydrogen-bonding interaction between the N–H group of TPU and the carboxyl group of PPC. The peak for C=O group of blends become broader, which due to the shift of C=O groups in pure PPC and TPU is different. The wavenumber of C=O groups in TPU is lower than which in PPC.

### 2.2. Morphology Observation

**Figure 2:**
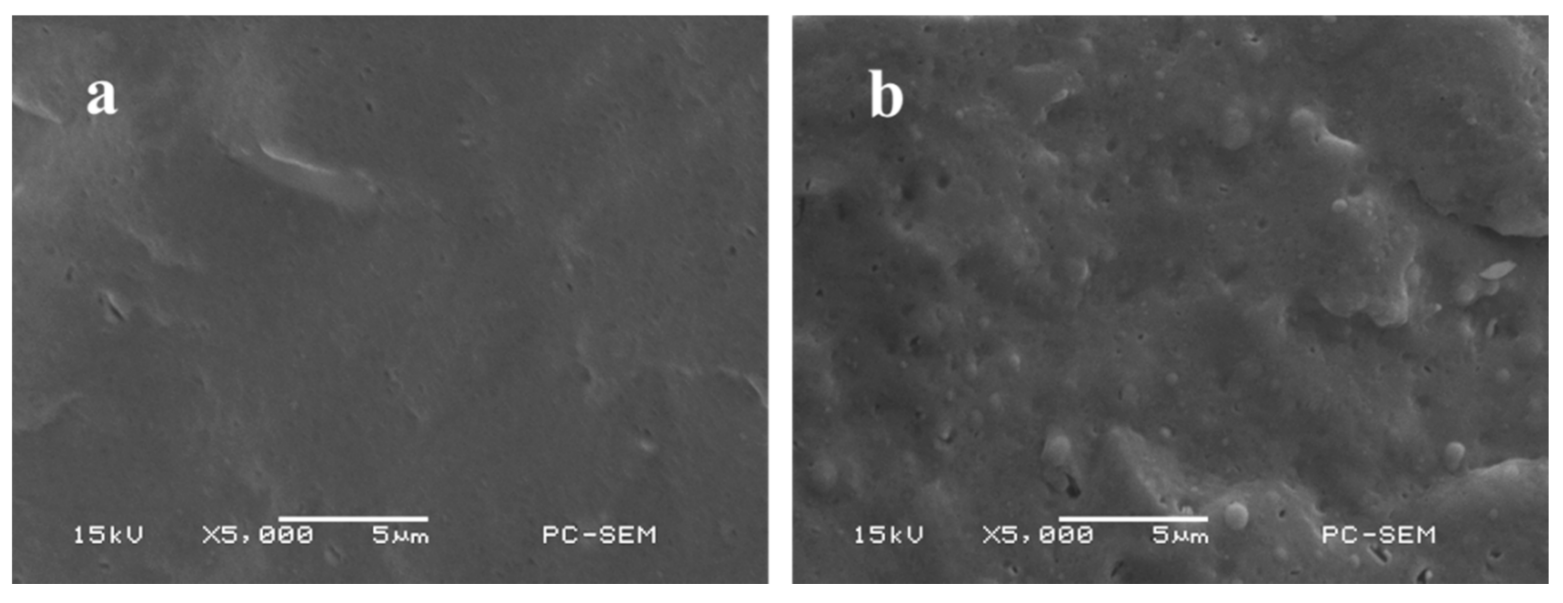
SEM micrographs of PPC and PPC/TPU blends. (**a**) Neat PPC, (**b**) PPC/10% TPU.

Figure 2 represents the typical SEM micrographs of cryogenically fractured surfaces of the pure PPC and the PPC/TPU blends with various TPU contents. It can be seen that the pure PPC shows a smooth and uniform surface. For PPC/TPU blends, TPU particles are well dispersed in PPC matrix in fine droplets. The interfaces between PPC and TPU are fuzzy, demonstrating the good interfacial adhesion between suspended TPU particles and PPC matrix. This indicates the improved compatibility between PPC and TPU and which owe to the intermolecular hydrogen-bonding formation. This result is quite different from Huang’s work and they reported that PLA is incompatible with TPU, although PLA also has carboxyl groups [24].

### 2.3. Thermal Behaviors

**Figure 3:**
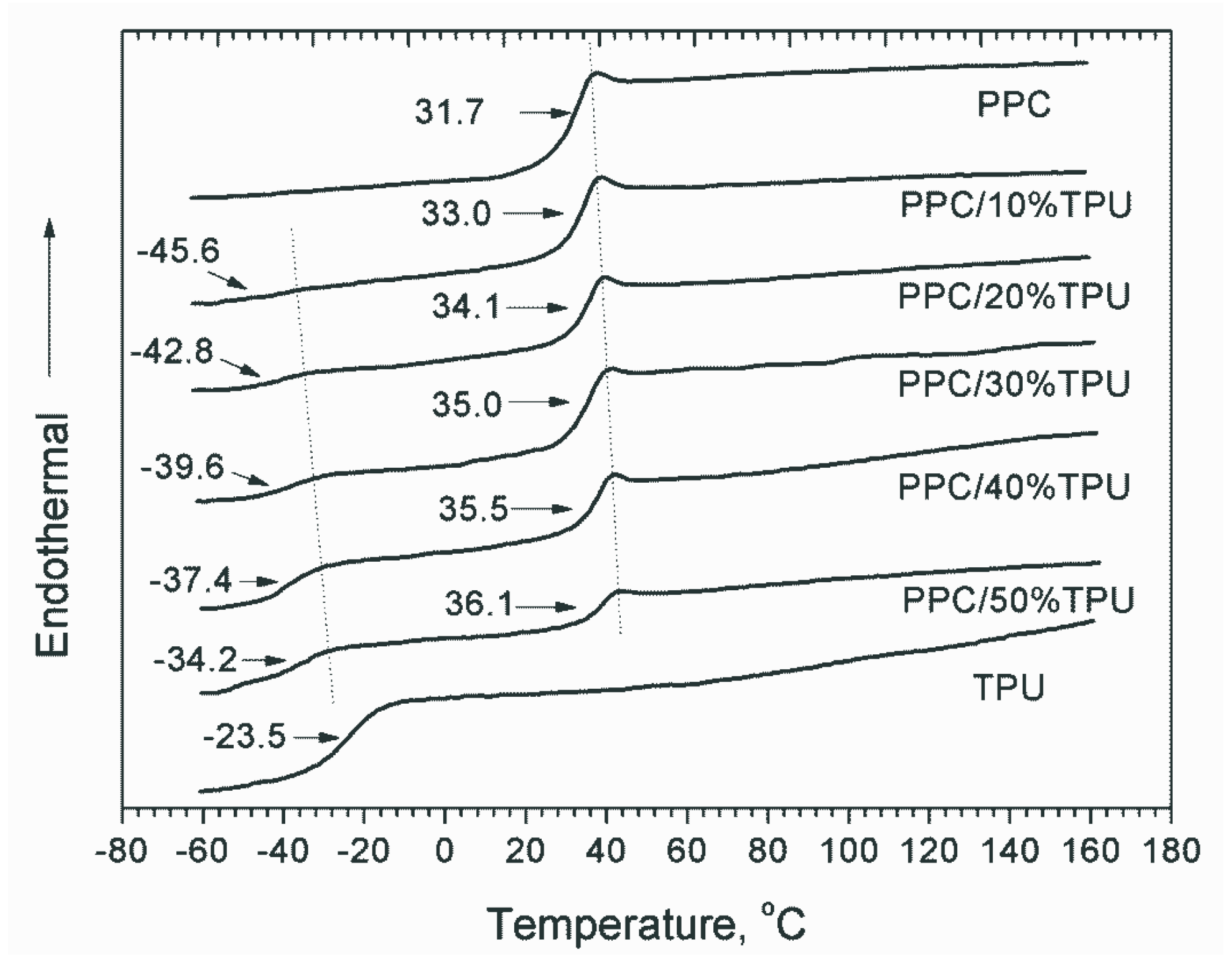
DSC curves of neat PPC, neat TPU and PPC/TPU blends.

Figure 3 shows the DSC traces of the neat PPC, neat TPU and PPC/TPU blends recorded on the second heating step. The soft segment of TPU has a glass transition temperature (Tg) of −23.5 °C, while there is no obvious glass transition for the hard segment of TPU. The Tg of PPC increases with increasing TPU content. The value reaches 36.1 °C with the addition of 50 wt % TPU, while that of neat PPC is 31.7 °C. The increasing of PPC’s Tg is presumably due to the hydrogen-bonding as indicated by FTIR investigation. Physical-crosslinking between PPC and TPU molecular chains greatly constrain the molecular movement of PPC matrix. On the contrary, the Tg value for soft segment of TPU decreases with increasing PPC content. It declines to about −34.2 °C with the addition of 50 wt % PPC. When PPC component increases to 90 wt %, the Tg value of TPU further decreases to as low as −45.6 °C. This is because the strong hydrogen-bonding between carbonyl of soft segment and the urothen groups of the TPU hard segment. The strong hydrogen-bonding decreases in turn constraining of soft segment of TPU, resulting in the decrease of Tg.

**Figure 4:**
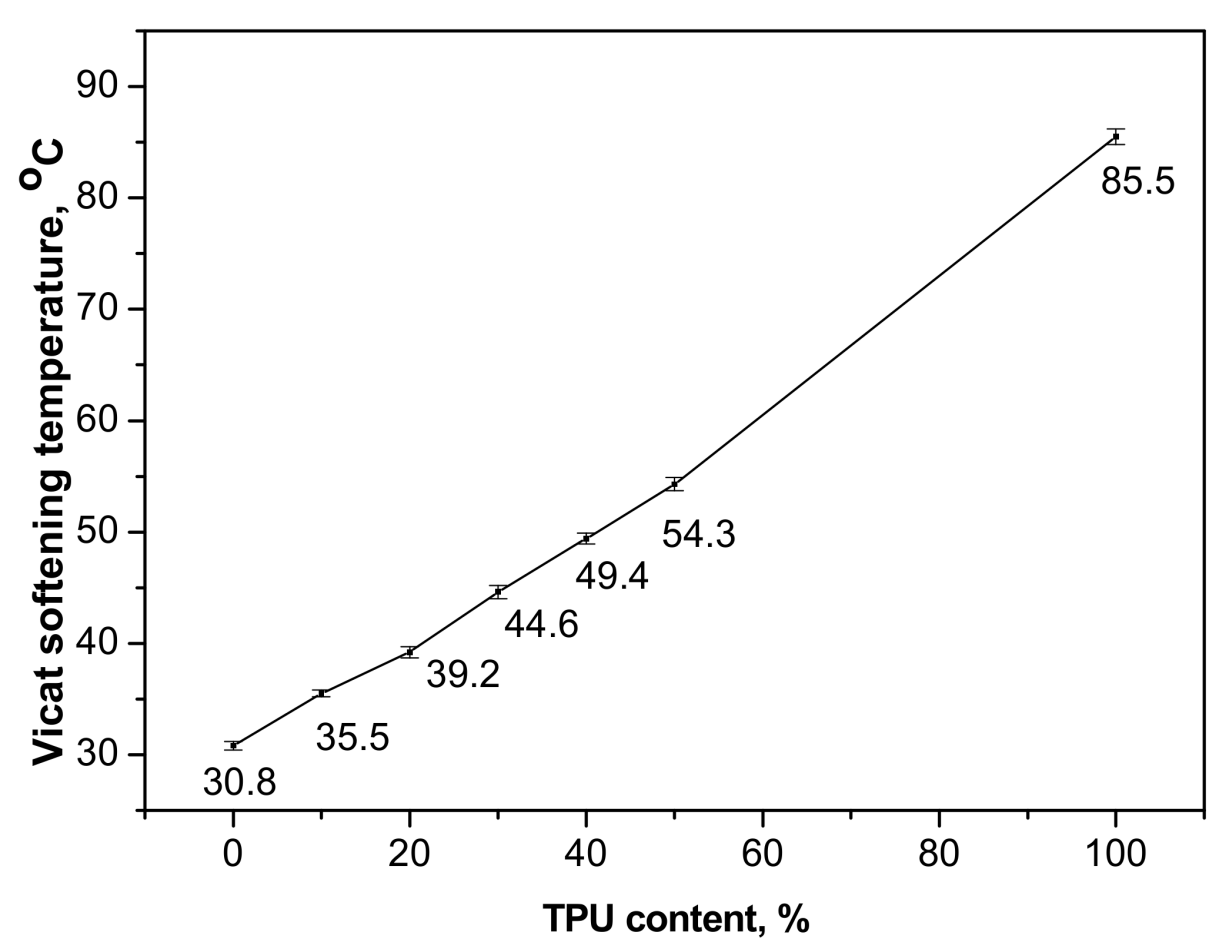
Effect of TPU content on Vicat softening temperatures of PPC/TPU blends.

The Vicat softening temperatures of the neat PPC, neat TPU and PPC/TPU blends are plotted in Figure 4. The Vicat temperatures shift to higher value with the incorporation of TPU. The value increases about 5 °C with every 10 wt % TPU addition. The improvement in thermal stability of PPC will certainly broaden the practical application of PPC.

### 2.4. Mechanical Properties

**Figure 5:**
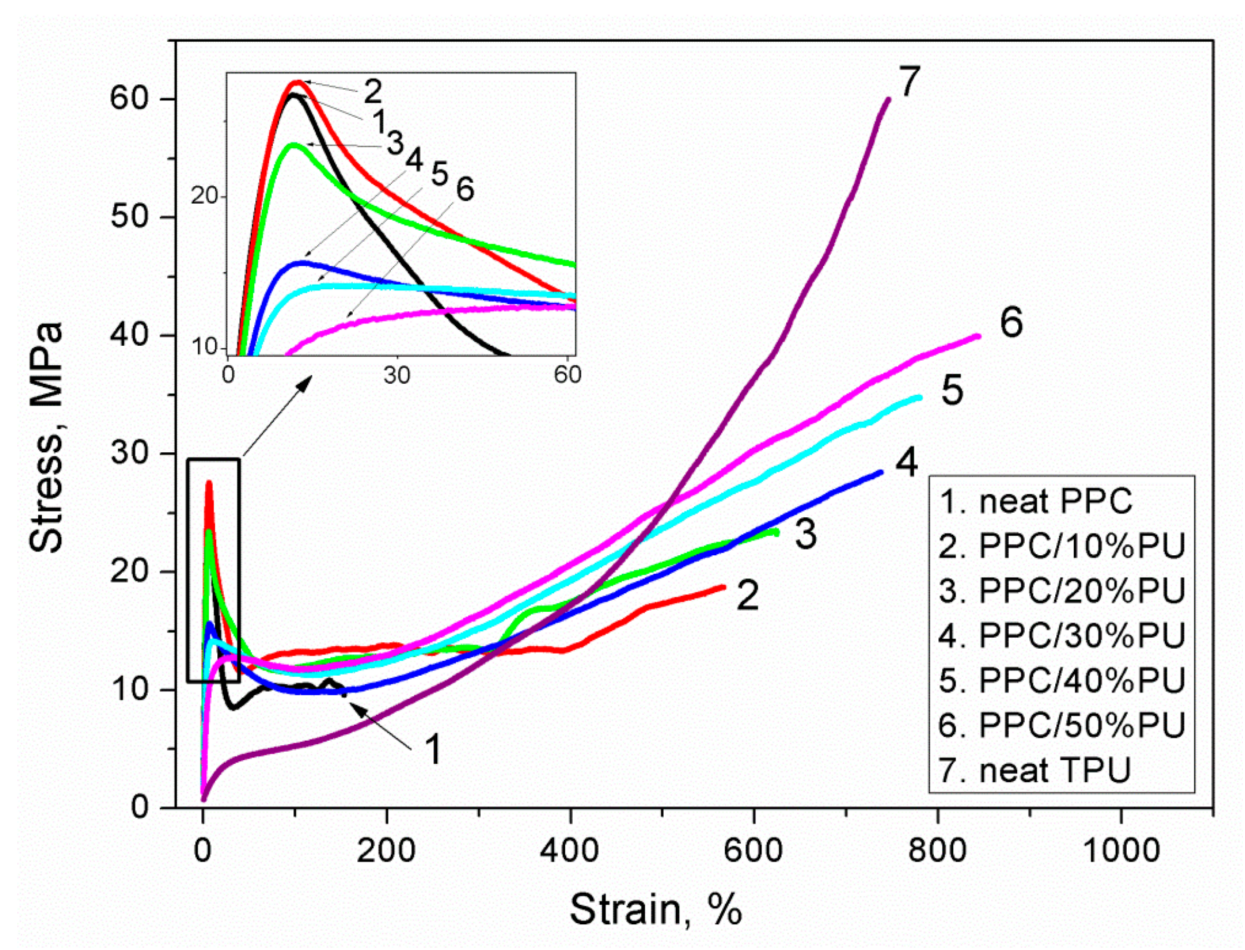
Stress-strain curves of PPC/TPU blends with different TPU contents.

The stress-strain curves for the neat PPC, neat TPU and PPC/TPU blends are given in Figure 5. It is apparent that TPU has a low modulus but high strain and stress at break. All PPC/TPU blends exhibit yield characteristics, nevertheless, PPC shows the lowest elongation at break compared with other PPC/TPU blends. The yield strength for each PPC/TPU blend is higher than that of neat PPC, indicating the formation of the hydrogen-bonding between PPC matrix and TPU particles. The addition of TPU significantly changes the tensile behavior of the PPC, especially the enhancement of elongation at break.

The yield stress, stress at break, elongation at break and tensile energy to break values of neat PPC, neat TPU and PPC/TPU blends are listed in Table 1. It can be seen that the stress at break, elongation at break increase dramatically due to the hydrogen-bonding between PPC and TPU of the blends. Many reports have disclosed that the yield stress of blends decrease with the addition of TPU [20,22,25]. In this work, however, the yield stress of PPC/10%TPU blend is little higher than that of the neat PPC due to the hydrogen-bonding. Because of the lower modulus of TPU, the yield stress of PPC/TPU blends decreases with increasing TPU. Moreover, both elongation at break and tensile energy to break increase sharply with the addition of TPU. It is interesting to note that the PPC/TPU blend with only 10 wt % TPU exhibits a very high elongation at break of 566.2%, together with a little increase of yield stress. The tensile energy increases up to about 5 times for PPC/10%TPU blend and to more than 11 times for PPC/50%TPU blend. PPC/40%TPU and PPC/50%TPU blends even show higher tensile energy and strain to break than that of neat TPU. The results demonstrate that both toughness and tensile strength of PPC can be improved by the simply introduction of TPU.

**Figure 6:**
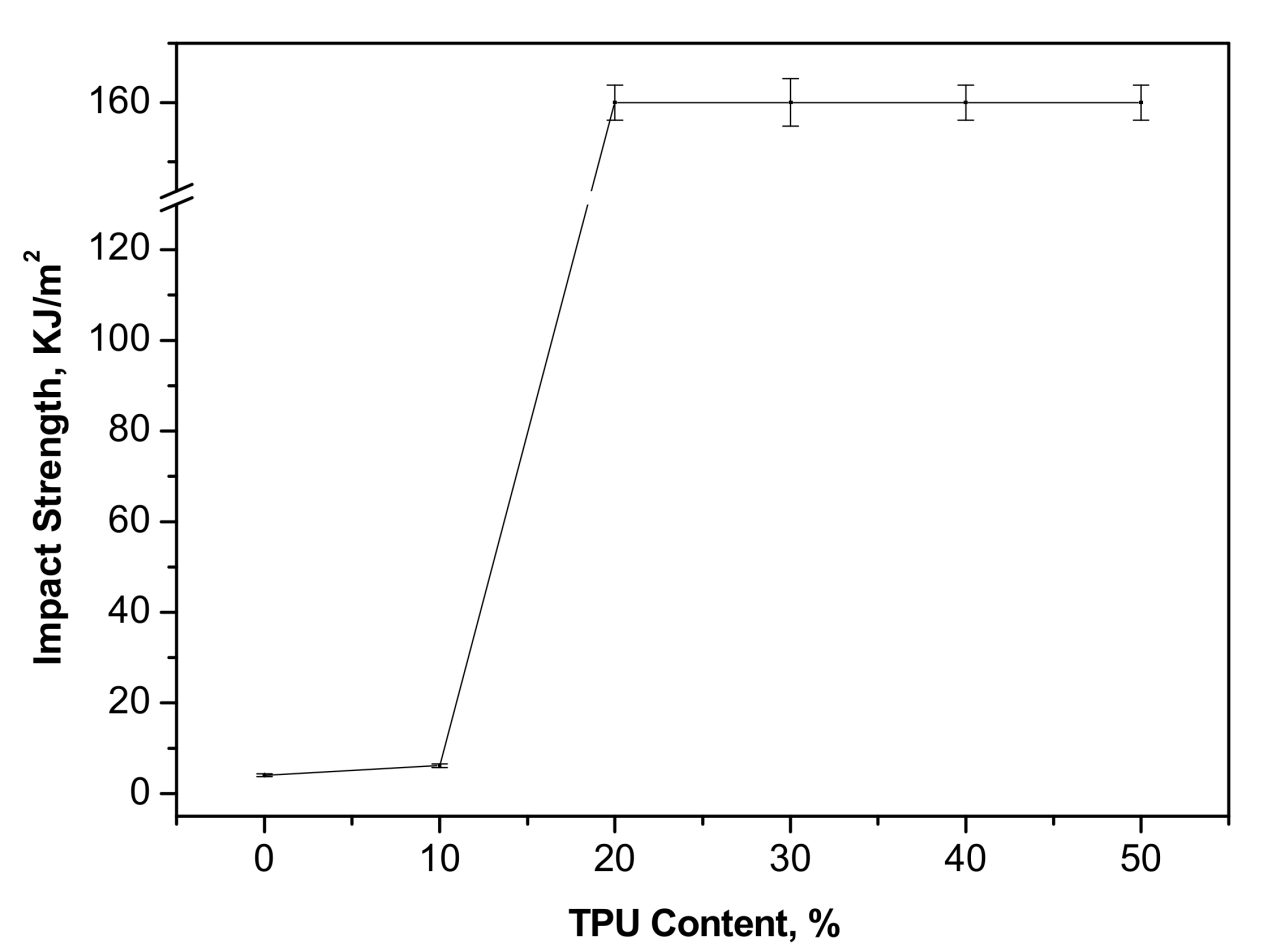
Impact strength of neat PPC and PPC/TPU blends as a function of TPU contents.

The charpy impact strength of the neat PPC and PPC/TPU blends as a function of TPU content and is shown in Figure 6. The impact strength of the PPC/TPU blends increases slightly with the addition of 10 wt % TPU. The impact strength increases dramatically with further increasing TPU content. Some of the impact specimens cannot be broken completely during impact testing. The PPC/TPU blends exhibit a brittle-ductile transition behavior when 20 wt % TPU added because the maximum impact strength of our impact tester is 125 kJ/m^2^ for standard specimen according to ASTM D256-05. These demonstrate the effective improvement of impact strength of PPC by the incorporation of TPU.

### 2.5. Toughening Mechanism

**Figure 7:**
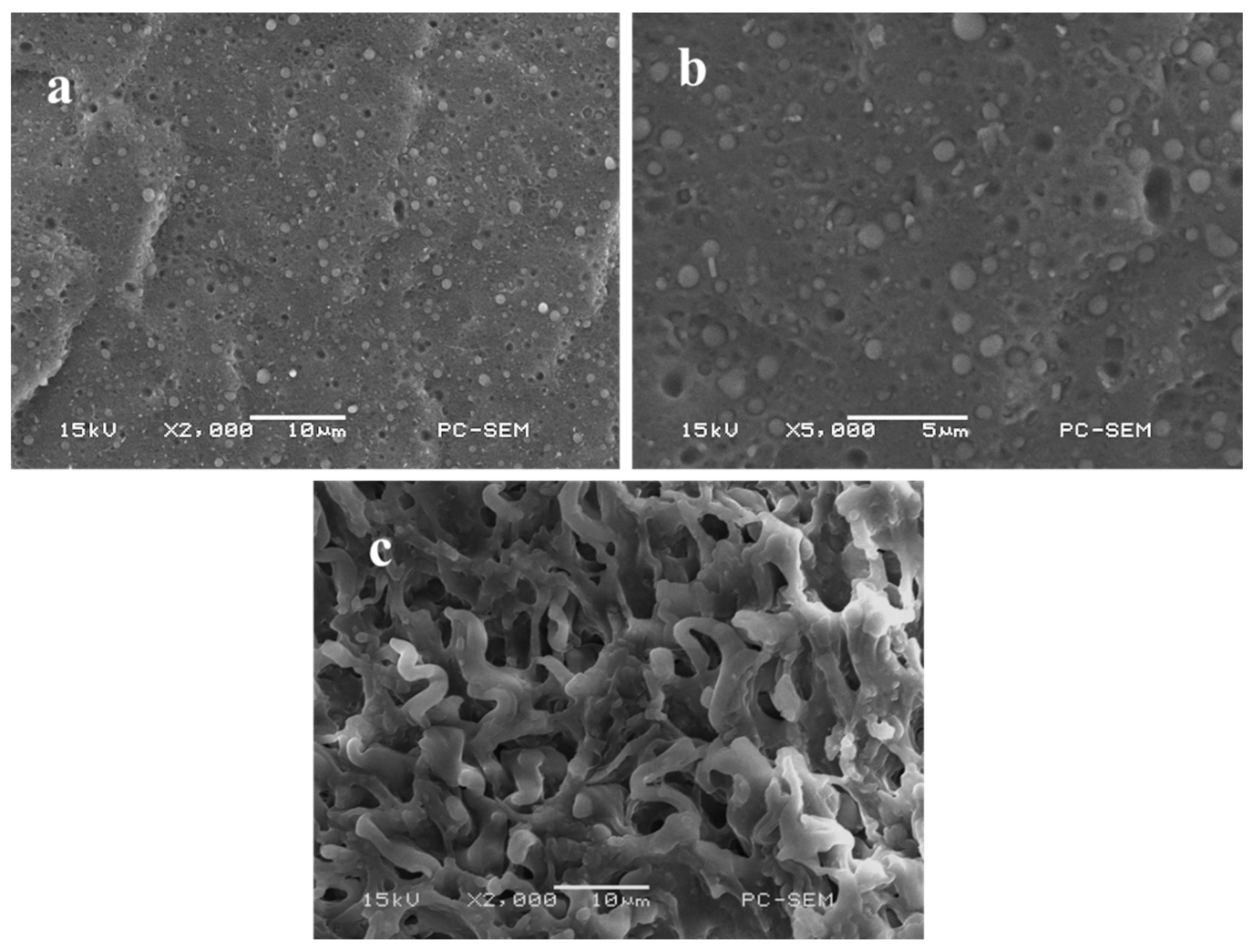
SEM micrographs of the impact fractured surfaces of PPC/TPU blends: (**a**) PPC/10%TPU, (**b**) enlarge view of a, (**c**) PPC/30%TPU.

The results of tensile and impact experiments show that the highly effective toughness of TPU on PPC, especially for the PPC/TPU blends with more than 20 wt % TPU. Figure 7 shows the micrographs of the impact fractured surfaces of PPC/TPU blends. According to neat PPC (Figure 2a), with the addition of 10 wt % TPU, the fractured surface seems very roughness (Figure 7a). From the enlarged image (Figure 7b), the suspended TPU droplets can be clearly seen with observable gaps between PPC matrix and TPU particles. The dispersed TPU particles play a crucial role as stress concentration point within PPC matrix, where the deformation is much easier to happen than other areas within PPC/TPU blends. TPU particles serve as the plasticizing deformation phase because of its high elasticity. The deformation can absorb impact energy, therefore, the impact strength of PPC/10%TPU blend increases about 50 wt % compared with that of neat PPC. The PPC/TPU blends tend to become a tough failure with increasing TPU content. The dispersed TPU particles, as a large number of stress concentration points within PPC matrix, initiate crazing and shear banding, thus, absorb a large number of impact energy and impede crack growth under a strong external shock. PPC/30%TPU blend shows a typical tough fracture (Figure 7c). It can be seen that the cavitation and extensive plastic deformation of both PPC matrix and suspended TPU particles, implying that shear yielding is more predominant than crazing of PPC matrix and TPU particles.

**Figure 8:**
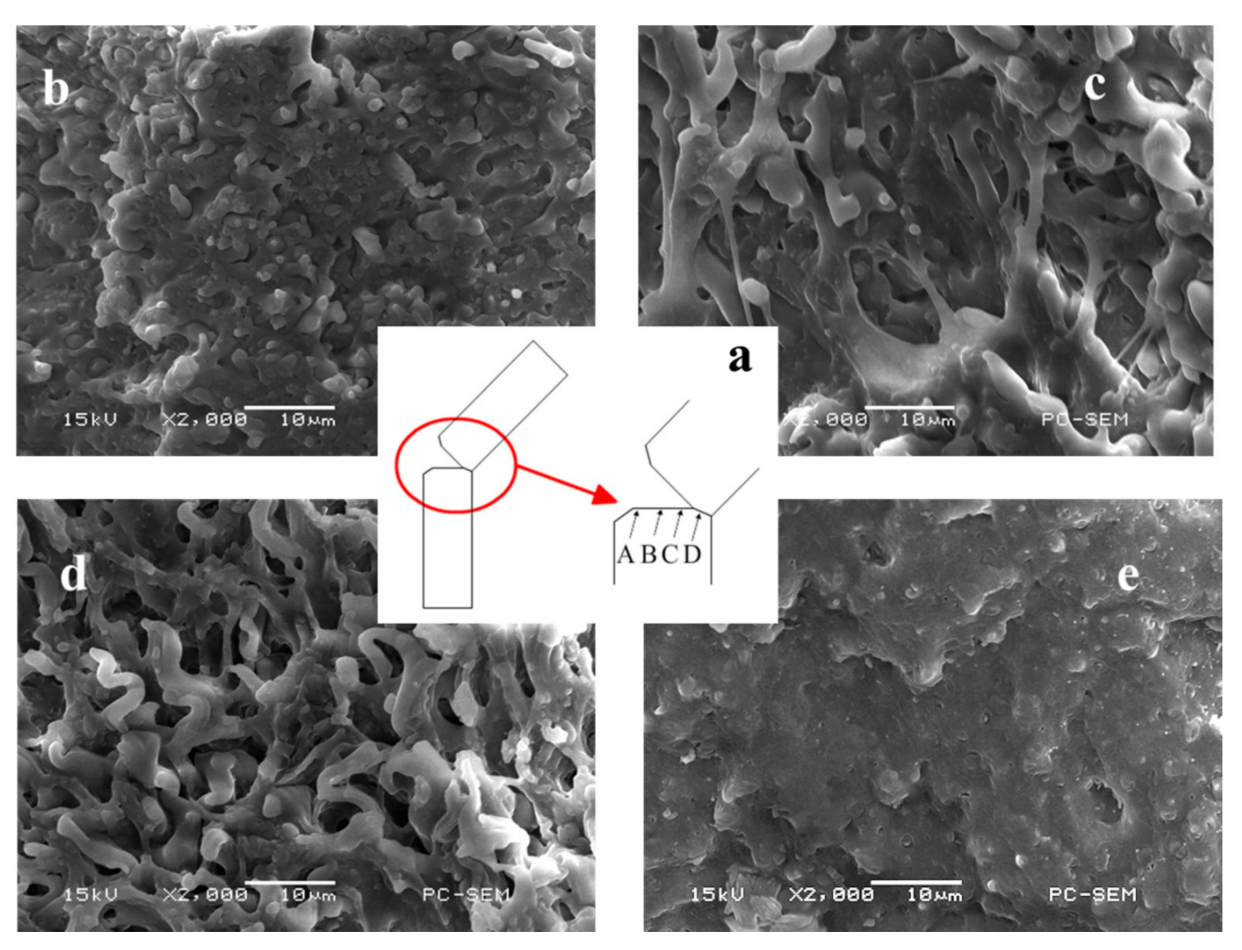
SEM micrographs of different regions on the impact fractured surface of the PPC/30%TPU sample. (**a**) Different regions of the impact fractured surface, (**b**–**e**) correspond to A–D in a, respectively.

In order to investigate the toughening mechanism, the different regions on the fractured surface of the impact fractured PPC/30%TPU blend sample is examined under SEM (Figure 8). The sample was not cracked completely and the cryogenically fractured surface along the impact direction (Figure 8a). Figure 8b–d shows different deformation states during impact testing. In the beginning of impact testing, the cracks were easily induced because of the high impact velocity and the stress concentration near the notch. The sample started to fracture immediately without large yielding as depicted in Figure 8b. Many voids around TPU particles were generated and some TPU particles showed small deformation and tended to be pulled out of PPC matrix. Thereafter, the impact energy was dissipated and the impact velocity decreased slightly. As shown in Figure 8c, further deformation of TPU particles can be seen clearly in point B. The PPC matrix around TPU particles deformed much obviously with shear yielding. This process absorbed the most energy of the impact fracture. As the impact velocity decreased sequentially, the sample showed again a tough failure in point C as shown in Figure 8d. The toughing mechanism can be explained as follows. First, TPU particles decrease the yield stress of PPC matrix following to generate shear yielding. Secondly, the PPC matrix deforms before cracking due to the decrease of impact velocity, which is attributed to the impact energy absorption from point A to point B. The micrograph of the freeze-fractured surface of the impact unfractured region is shown in Figure 8e. From this figure, it can be seen that the fractured surface is much smoother than other regions, only a few gaps between PPC matrix and TPU particles can be observed. Because the most of applied impact energy is dissipated before point D, there is not enough impact energy to destroy the blend sample with high TPU contents.

### 2.6. Dynamic Mechanical Analysis

The dynamic mechanical analysis (DMA) can be used to characterize the secondary motion and properties of polymer molecules at low temperature [26], except for the characterization of the polymerized glass transition temperature.

**Figure 9:**
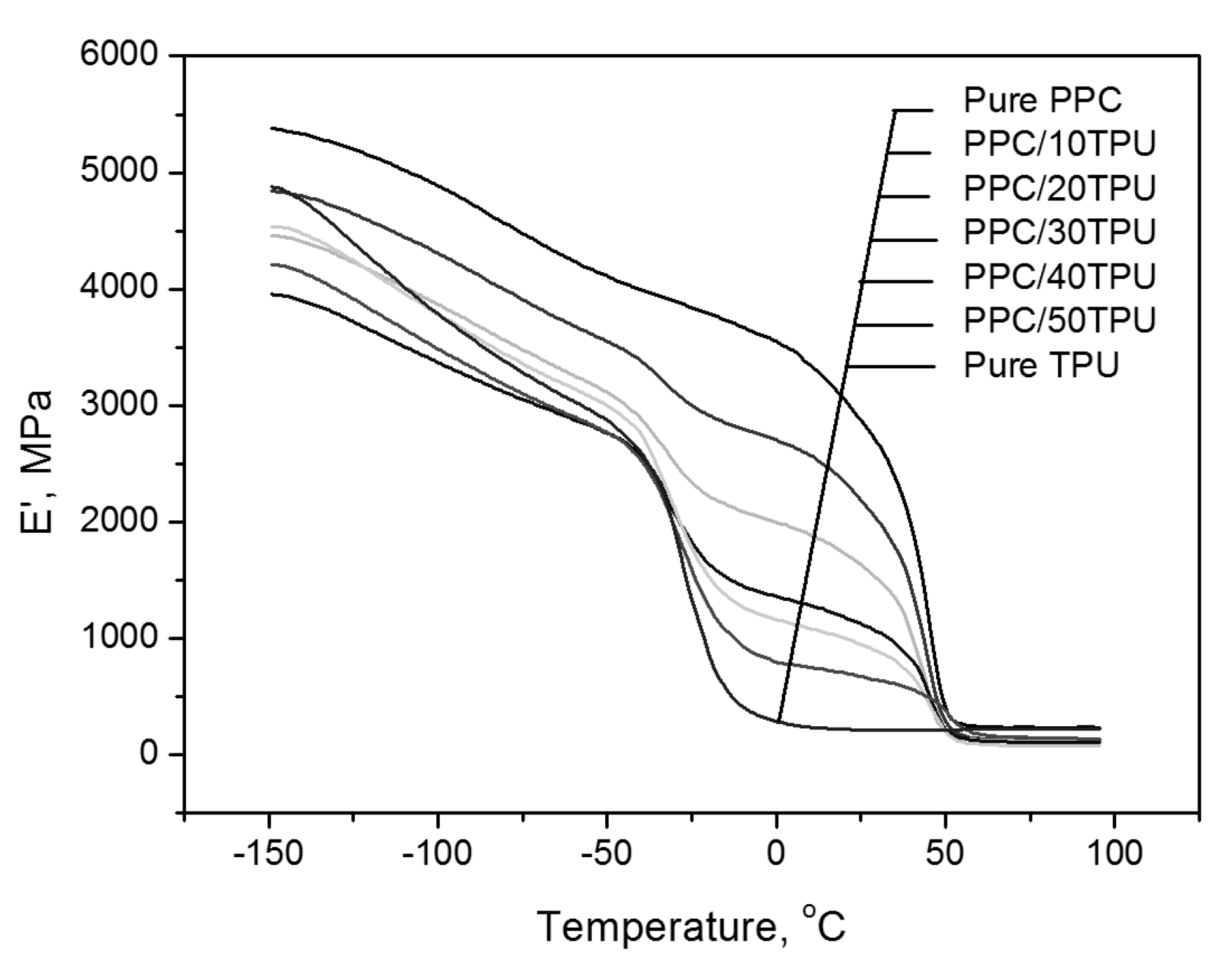
Storage modulus versus temperature for PPC, TPU and PPC/TPU blends.

The plots of storage modulus as a function of temperature for PPC, TPU and PPC/TPU are showed in Figure 9. It can be seen that in addition to a stress relaxation between 10 and 50 °C, pure PPC also has a secondary transition in the low temperature range of −100°C to −50 °C. Similarly, in addition to glass transition between −50 °C and 0 °C, pure TPU has a large secondary transition between −150 °C and −60 °C, which is important to the excellent flexibility and good performance at low temperature. Where When the TPU is in a highly elastic state, the storage modulus of the blends material gradually decreases as the TPU content increases, which indicates that the flexibility of the blends material is improved, which is one of the reasons why stretching and impact toughness is getting better with the increase of the TPU content.

**Figure 10:**
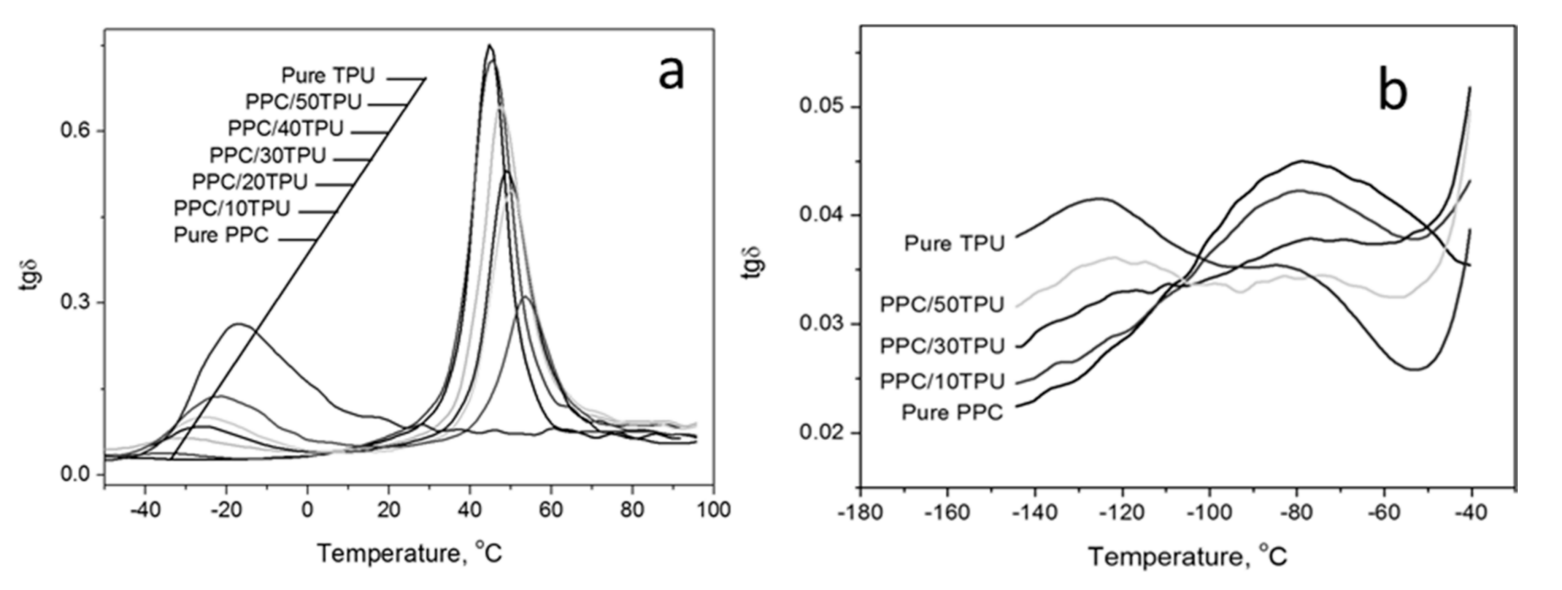
tg δ versus temperature for PPC, TPU and PPC/TPU blends: (**a**) higher temperature, (**b**) lower temperature.

Figure 10a shows the tg δ curves of PPC, TPU and their blends in the higher temperature region. The two peaks in the curve represent the glass transitions of PPC and TPU, respectively. The high temperature is according to the glass transition of PPC. The lower temperature is according to the glass transition of TPU. With the increase of TPU content, the corresponding peak of PPC becomes lower and lower and the corresponding peak of TPU becomes higher and higher. The corresponding value of the peak position is the glass transition temperature of the material and which is listed in Table 2. For PPC, as the content of TPU increases, the glass transition temperature of PPC increases gradually, from 44.7 °C of pure PPC to 53.5 °C when 50% TPU is added and 8.8 °C is increased. For TPU, as the content of PPC increases, the glass transition temperature of TPU gradually decreases, which is consistent with the results obtained by DSC analysis.

It could be seen the tg δ curves of PPC, TPU and its blends in the lower temperature region in Figure 10b. It shows that both PPC and TPU have a β-transformation in the low temperature region and with the increase of TPU content, the β-transition peak of PPC in the blends material is becoming smaller, while the β-transition of TPU is more and more obvious. It indicates that as the TPU content increases, the high-temperature β-transition in the blends slowly disappears and replaced by a lower-temperature β-transition. The shift of β to low temperature means that the low temperature toughness of the material is improved. Therefore, the brittle-ductile transition temperature of the alloy material gradually decreases with the increase of the TPU content, showing better flexibility at low temperature.

## 3. Materials and Methods

### 3.1. Materials

The high molecular weight Poly(propylene carbonate) (PPC), with a number-average molecular weight (Mn) of 109,000 Da and a polydispersity (PD) of 1.91, was provided by Tian’guan Enterprise Group Co. (Henan, China). Polyester-based TPU (grade S685AL, density = 1.2 g/cm^3^), was obtained from Kin Join Co., Ltd. (Taiwan). Both PPC and TPU pellets were dried in a vacuum oven for 24 h at 80 °C before blending.

### 3.2. Preparation of PPC/TPU Blends

The PPC/TPU blends with weight ratios of 100/0, 90/10, 8/20, 70/30, 60/40, 50/50 and 0/100 were prepared in a Haake Rheomix RT 600 mixer (Haaker, Germany). The mixing was carried out at 160 °C with a rotary speed of 40 rpm for 7 min. The prepared blends were then melt pressed at 170 °C into standard dumbbell tensile bars (ASTM D638) and standard V-shaped notched impact bars (ASTM D256-05).

### 3.3. Fourier Transform Infrared (FTIR) Spectra

FTIR spectra were recorded on a Perkin-Elmer FTIR-100 spectrometer (Perkin-Elmer, Waltham, MA, USA). Samples were first melt pressed to thin film and then scanned with wavenumbers from 4000 to 400 cm^−1^ with a resolution of 4.0 cm^−1^.

### 3.4. Scanning Electron Microscopy (SEM) Observation

The morphologies of the blends were observed using SEM (Jeol JSM-6380, JEOL, Tokyo, Japan). All of the Specimens were fractured in liquid nitrogen; the fracture surface was then coated with a thin layer of gold. The fracture surface after impact tests were also observed by the same SEM apparatus.

### 3.5. Differential Scanning Calorimetry (DSC) Investigation

The samples were scanned with differential scanning calorimetry (DSC, Netzsch 204, Selb, Bavaria, Germany). The temperature was initially heated from room temperature to 190 °C at a rate of 10 °C/min, maintained at 190 °C for a period of 3 min to eliminate previous thermal history, then cooled down to −100 °C at 10 °C/min, maintained at −100 °C for 3 min. The samples were subsequently scanned back to 190 °C with a heating rate of 10 °C/min. All the scanning processes were under a protective atmosphere of N2.

### 3.6. Vicat Softening Temperature

The Vicat softening temperatures of the blends were measured by a Vicat tester (New SANS, Shen Zhen, China) at 10 N load and heating rate of 50 °C/h according to ASTM D 1525. Six specimens have been examined for each blend and average value was reported accordingly.

### 3.7. Static Tensile Properties

The static tensile properties were investigated using a universal mechanical testing machine (New SANS) at 25 °C with a relative humidity of 50 ± 5%. The crosshead speed was set at 50 mm/min. Five specimens of each sample were measured and the average results were recorded. All the samples were conditioned at 25 °C and 50% RH for 24 h before testing.

### 3.8. Charpy Impact Strength

The Charpy impact strengths of notched specimens were determined using a Charpy impact tester (SANS ZBC-4B, MTSSANS, Eden Prairie, Minnesota, USA) at 25 °C and 50% RH according to ASTM D256-05. The maximum velocity of the pendulum is 2.9 m/s, generating impact energy of 4 J. Each blend of five specimens was measured and the average results were recorded. All the samples were conditioned for 24 h at 25 °C and 50% RH before testing.

### 3.9. Dynamic Mechanical Analysis

The dynamic mechanical analysis of the material was carried out by a dynamic mechanical analyzer (DMA, model 242D, Netzsch, Selb, Germany). The test was conducted in a single cantilever vibration mode. The vibration frequency was 3.3 Hz and the amplitude was 100 μm. The size of the specimen is 35 mm × 10 mm × 1 mm and the temperature is set from −150 °C to 150 °C, with a heating rate of 5 °C/min.

## 4. Conclusions

PPC/TPU blends can be readily prepared by melt blending. Experimental results indicate the existence of intermolecular hydrogen-bonding between the carbonyl groups of PPC and the urethane groups of TPU. The SEM examination and thermal analysis of the PPC/TPU blends show a compatible blend system. The incorporation of TPU can obviously enhance the thermal stability and broaden the application temperature of PPC according to DSC and Vicat softening temperature investigation. Moreover, the toughness of the blends increases dramatically with increasing TPU content. The blend exhibits a brittle-ductile transition temperature at about 25 °C with the addition of 20 wt % TPU. Based on the experiment results, we have proposed a toughening mechanism of TPU for PPC matrix. The shear yielding occurs in both suspended TPU particles and PPC matrix, which accounts for the sharp increase in the mechanical properties of PPC.

## Figures and Tables

**Table 1:** Mechanical property of PPC/TPU blends with different TPU contents.

TPU Content, %	Yield Stress, MPa	Stress at Break, MPa	Elongation at Break, %	Tensile Energy to Break, MJ/m^3^	Stress at 100%, MPa	Stress at 200%, MPa	Stress at 300%, MPa
0	26.5 ± 1.2	9.60 ± 0.6	180 ± 20	17.1 ± 3	10.3 ± 0.3	-	-
10	27.6 ± 0.9	18.7 ± 0.7	570 ± 30	82.2 ± 9	13.2 ± 0.2	13.8 ± 0.2	13.3 ± 0.3
20	23.5 ± 0.8	23.2 ± 0.4	620 ± 40	103 ± 7	11.9 ± 0.4	12.9 ± 0.3	13.5 ± 0.2
30	18.6 ± 0.5	27.5 ± 0.7	750 ± 50	124 ± 11	9.9 ± 0.2	10.7 ± 0.2	13.3 ± 0.2
40	14.3 ± 0.3	33.5 ± 2	780 ± 30	159 ± 9	11.4 ± 0.4	12.4 ± 0.3	15.2 ± 0.4
50	12.8 ± 0.5	40.0 ± 3	840 ± 50	195 ± 14	11.8 ± 0.2	12.9 ± 0.4	16.4 ± 0.4

**Table 2:** Glass transition temperature for PPC, TPU and their blends from dynamic mechanical analysis (DMA).

TPU Content, %	0	10	20	30	40	50	100
Tg (PPC), °C	44.7	45.4	47.5	48.9	50.2	53.5	-
Tg (TPU), °C	-	−35.9	−31.6	−26.3	−24.8	−22.1	−16.8

## References

[1] Inoue S., Tsuruta T., Kobayash M., Koinuma H. (1972). Reactivities of Some Organozinc Initiators for Copolymerization of Carbon Dioxide and Propylene Oxide. Die. Makromol. Chem..

[2] Darensbourg D. (2007). Making Plastics from Carbon Dioxide: Salen Metal Complexes as Catalysts for the Production of Polycarbonates from Epoxides and CO_2_. J. Chem. Rev..

[3] Luinstra G.A. (2008). Poly(Propylene Carbonate), Old Copolymers of Propylene Oxide and Carbon Dioxide with New Interests: Catalysis and Material Properties. Polym. Rev..

[4] Inoue S., Tsuruta T. (1975). Synthesis and thermal degradation of carbon dioxide-epoxide copolymer. Appl. Polym. Symp..

[5] Ren W.M., Zhang X., Liu Y., Li J.F., Wang H., Lu X.B. (2010). Highly Active, Bifunctional Co(III)-Salen Catalyst for Alternating Copolymerization of CO_2_ with Cyclohexene Oxide and Terpolymerization with Aliphatic Epoxides. Macromolecules.

[6] Song P.F., Xiao M., Du F.G., Wang S.J., Gan L.Q., Liu G.Q., Meng Y.Z. (2008). Synthesis and properties of aliphatic polycarbonates derived from carbon dioxide, propylene oxide and maleic anhydride. J. Appl. Polym. Sci..

[7] Seong J.E., Na S.J., Cyriac A., Kim B.W., Lee B.Y. (2003). Terpolymerization of CO_2_ with Propylene Oxide and ε-Caprolactone Using Zinc Glutarate Catalyst. Macromolecules.

[8] Zeng S.S., Wang S.J., Xiao M., Han D.M., Meng Y.Z. (2011). Preparation and properties of biodegradable blend containing poly (propylene carbonate) and starch acetate with different degrees of substitution. Carbohydr. Polym..

[9] Ge X.C., Li X.H., Zhu Q., Li L., Meng Y.Z. (2004). Preparation and properties of biodegradable poly(propylene carbonate)/starch composites. Polym. Eng. Sci..

[10] Tao J., Song C.J., Cao M.F., Hu D., Liu L., Liu N., Wang S.F. (2009). Thermal properties and degradability of poly(propylene carbonate)/poly(β-hydroxybutyrate-*co*-β-hydroxyvalerate) (PPC/PHBV) blends. Polym. Degrad. Stab..

[11] Yang D.Z., Hu P. (2008). Miscibility, crystallization, and mechanical properties of poly(3-hydroxybutyrate) and poly(propylene carbonate) biodegradable blends. J. Appl. Polym. Sci..

[12] Ma X.F., Yu J.G., Wang N. (2006). Compatibility characterization of poly (lactic acid)/poly (propylene carbonate) blends. J. Polym. Sci. Part B Polym. Phys..

[13] Pang M.Z., Qiao J.J., Jiao J., Wang S.J., Xiao M., Meng Y.Z. (2008). Miscibility and properties of completely biodegradable blends of poly(propylene carbonate) and poly(butylene succinate). J. Appl. Polym. Sci..

[14] Jiao J., Wang S.J., Xiao M., Xu Y., Meng Y.Z. (2007). Processability, property, and morphology of biodegradable blends of poly (propylene carbonate) and poly (ethylene-*co*-vinyl alcohol). Polym. Eng. Sci..

[15] Simmons A., Hyvarinen J., Poole-Warren L. (2006). The effect of sterilisation on a poly (dimethylsiloxane)/poly (hexamethylene oxide) mixed macrodiol-based polyurethane elastomer. Biomaterials.

[16] Lebedev E.V., Ishchenko S.S., Denisenko V.D., Dupanov V.O., Privalko E.G., Usenko A.A., Privalko V.P. (2006). Physical characterization of polyurethanes reinforced with the in situ-generated silica-polyphosphate nano-phase. Compos. Sci. Technol..

[17] Xian W.Q., Song L.N., Liu B.H., Ding H.L., Li Z., Cheng M.P., Ma L. (2018). Rheological and mechanical properties of thermoplastic polyurethaneelastomer derived from CO_2_ copolymer diol. J. Appl. Polym. Sci..

[18] Lu Q.W., Macosko C.W., Lu Q.W., Macosko C.W. (2004). Comparing the compatibility of various functionalized polypropylenes with thermoplastic polyurethane (TPU). Polymer.

[19] Feng F., Ye L. (2011). Morphologies and mechanical properties of polylactide/thermoplastic polyurethane elastomer blends. J. Appl. Polym. Sci..

[20] Li Y.J., Shimizu H. (2007). Toughening of polylactide by melt blending with a biodegradable poly (ether) urethane elastomer. Macromol. Biosci..

[21] Palanivelu K., Balakrishnan S., Rengasamy P. (2000). Thermoplastic polyurethane toughened polyacetal blends. Polym. Test..

[22] Palanivelu K., Sivaraman P., Reddy M.D. (2002). Studies on thermoplastic polyurethane toughened poly (butylene terephthalate) blends. Polym. Test..

[23] Peng S.W., Wang X.Y., Dong L.S. (2005). Special interaction between poly (propylene carbonate) and corn starch. Polym. Compos..

[24] Han J.J., Huang H.X. (2011). Preparation and characterization of biodegradable polylactide/thermoplastic polyurethane elastomer blends. J. Appl. Polym. Sci..

[25] Chang F.C., Yang M.Y. (1990). Mechanical fracture behavior of polyacetal and thermoplastic polyurethane elastomer toughened polyacetal. Polym. Eng. Sci..

[26] Borah J.S., Chaki T.K. (2011). Dynamic mechanical, thermal, physico-mechanical and morphological properties of LLDPE/EMA blends. J. Polym. Res..

